# The dermal deposits in the finger of the Holy Roman Emperor Charles I of Spain and V of Germany

**DOI:** 10.3389/fmed.2023.1287041

**Published:** 2024-01-12

**Authors:** Angel Fernandez-Flores, Eduardo Fonseca

**Affiliations:** ^1^Department of Cellular Pathology, Hospital El Bierzo, Ponferrada, Spain; ^2^Department of Cellular Pathology, Hospital de la Reina, Ponferrada, Spain; ^3^Department of Research, Institute for Biomedical Research of A Coruña (INIBIC), University of A Coruña (UDC), A Coruña, Spain; ^4^Department of Dermatology, Universitary Hospital of A Coruña, A Coruña, Spain

**Keywords:** Charles V, Yuste, malaria, gout, Zulueta, Paludism Charles V, Paludism

## Abstract

Charles I of Spain and V of Germany was one of the most prominent figures in Christendom. The vast empire ruled by the monarch extended across multiple continents. However, the final years of his life were overshadowed by depression and incapacitating joint degeneration, leading him to retire to the Monastery of Yuste in Cáceres, Spain. It was there that he contracted malaria, a disease that ultimately claimed his life. In this article, we evaluate the studies conducted on one of his mummified fingers to confirm the presence of malaria and investigate the cause of his joint degeneration, which was attributed to intense deposits of uric acid.

## 1 Introduction

Charles I of Spain and V of the Holy Roman Empire (1500–1,558) was formerly referred to as “Caesar” owing to the extensive expanse of his empire (1). During his reign, he unified the Crowns of Castile, which encompassed the Kingdom of Navarre, and Aragon. Additionally, he held the title of Holy Roman Emperor from 1,520 to 1,558. Inherited from his lineage were the Burgundian heritage, the Austrian territories (including the claim to the Imperial Throne), the Indies, Naples, and Sicily. This gave rise to the legendary notion that in his dominions, the sun never set.

Despite his remarkable vigor, the emperor faced severe health issues during the final days of his rule, leading to the suspension and postponement of significant military campaigns, some of which held great importance. This was the case with the unsuccessful attempt to conquer Metz in 1552. Subsequent military setbacks, combined with the emperor’s battle with depression, ultimately led to his abdication at the age of 56.

In this article, we aim to provide a historical context for the study of two of his most well-known illnesses. On one hand, the recurrent gout attacks that the Emperor suffered during the final period of his life, incapacitating him for the routine conduct of his daily activities. On the other hand, malaria, which ultimately led to his demise.

## 2 Materials and methods

For this work, we have turned to the following sources: PubMed has been consulted for texts related to existing scientific studies on the finger of Charles V. Regarding the Emperor’s biographical context, the primary biographical texts from recent decades have been consulted. The verbatim quotes included in the text and attributed to the Emperor have been primarily sourced from two different references.

Finally, the residence where the Emperor passed away in Yuste, Spain, has been visited.

## 3 The Emperor’s final period

The Emperor’s persistent health issues most likely undermined his strength to the extent of leading him to abdication. Nevertheless, the abdication did not occur in a single instance but rather took place in multiple stages (2). Out of all these stages, only the official abdication of complete sovereignty over the Netherlands was formally recognized (2).

**Figure 1:**
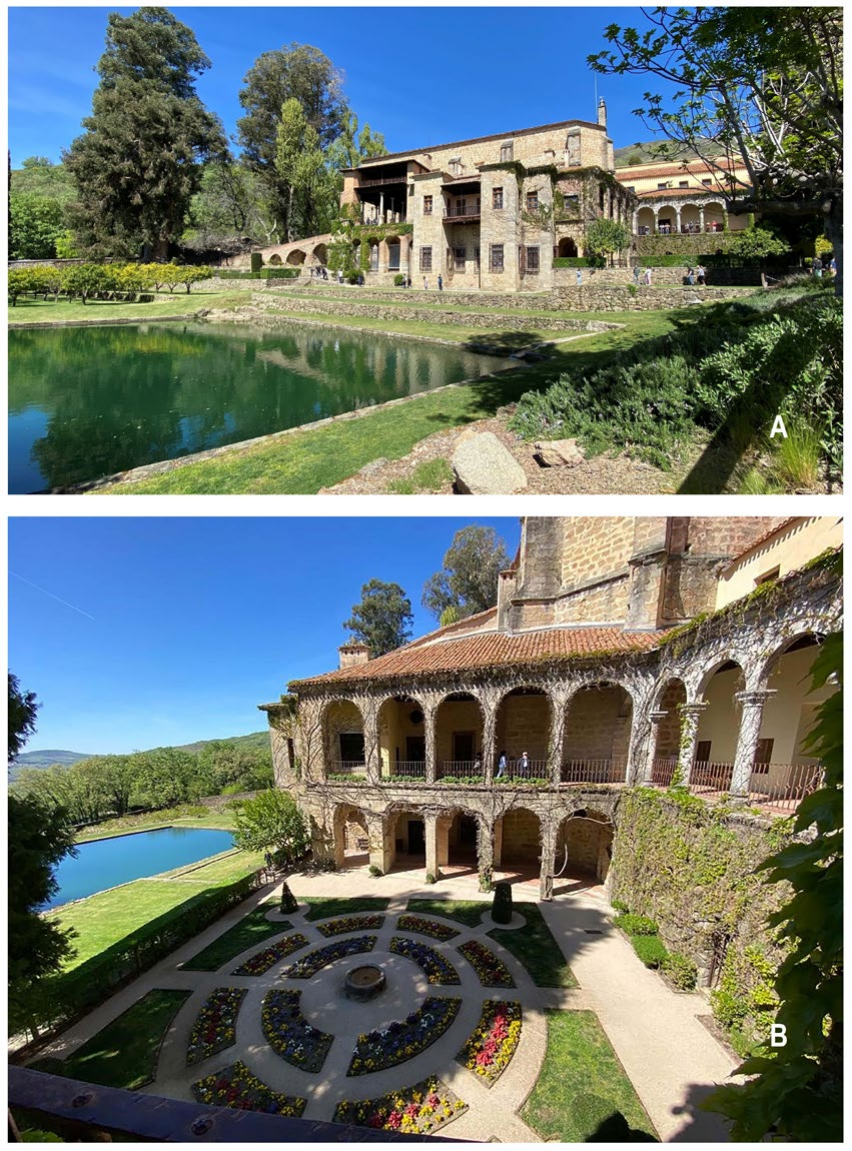
**(A)** An overall view of the Monastery of Yuste in Cáceres, Spain, where Emperor Charles V retired during his final days. The photograph prominently showcases the pond, designed by architect Juanelo Turriano, in the foreground (Photograph taken by the authors of the manuscript). **(B)** View of the balcony of the imperial residence. These rooms were divided into two levels: a warmer upper level for winter and a cooler lower level for summer (Photograph taken by the authors of the manuscript).

To overcome his depression, the Emperor embarked on an extensive retreat at the Monastery of Yuste (Cáceres, Spain) (Figures 1A,B), where he withdrew on February 3, 1557 (3). The selection of this retreat location had been made by the Emperor himself prior to his abdication. Carlos V opted for Yuste due to its affiliation with the Hieronymite order, which held his favor, and he conveyed this preference to his son Felipe (2). The journey to Yuste proved strenuous due to the treacherous roads, and the Emperor’s fragile mental state compounded the challenges (2).

During his residence in Yuste, Carlos V spent 20 months enduring numerous bouts of gout, which significantly impaired his mobility (2, 4). Nonetheless, he managed to maintain his daily routine, which included activities such as prayer, reading sacred texts, exploring history, botany, science, and participating in the daily liturgy (5–7). He also took solace in contemplating his collection of paintings, medals, maps, and navigation charts, as well as tending to his scientific instruments and timepieces. Furthermore, he enjoyed strolls through the gardens and indulged in fishing in one of the nearby ponds (2). These ponds were among the architectural modifications implemented prior to Carlos V’s retirement (2). The Emperor had requested specific architectural changes to the monastery’s premises and gardens, and it was the idea of the Italian engineer Juanelo Turriano to construct several ponds within the monastery’s gardens. While this architectural enhancement aimed to provide a refreshing atmosphere during sunny summer afternoons, it unintentionally resulted in a significant increase in the mosquito population in the area. In a recent study conducted by Tropical Medicine specialist Julián de Zulueta, a high density of Anopheles atroparvus mosquitoes was found in the vicinity of the Yuste monastery.

It is highly probable that the Anopheles mosquitoes were responsible for transmitting the malaria infection that afflicted the emperor, with symptoms starting to manifest in the summer of 1,558 (4). On August 31st, 1558, after dining outdoors, he confided to one of his physicians: ‘*I feel unwell, Doctor*’ (6, 8). Subsequently, the Emperor experienced recurring paroxysmal fevers, diagnosed as ‘*double tertian malignant fever*’, which were treated through practices such as bloodletting and enemas. For instance, on September 2nd, 10 ounces of blood were extracted from him, followed by intense thirst that he had to alleviate with vinegar and beer. Nevertheless, these efforts failed to halt the progressive decline in his health, as acknowledged by the emperor himself, who requested updates to his will. On September 2nd, initial bloodletting procedures were performed, yielding unsatisfactory results, and his condition further deteriorated by the 6th. Consequently, on September 9th, Charles V made the decision to draft an additional codicil to his Brussels testament of 1,554 (9).

**Figure 2:**
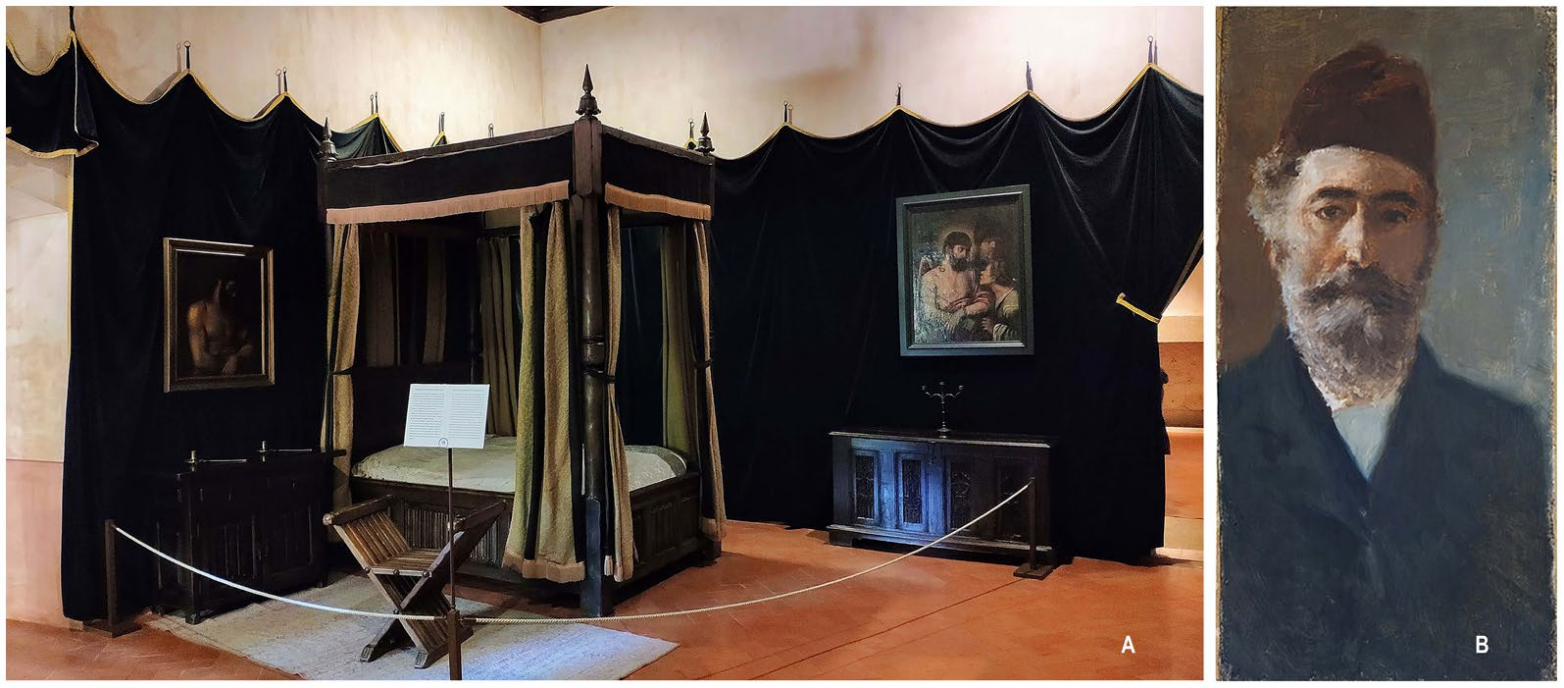
**(A)** Deathbed of the Emperor, located in the Monastery of Yuste, Cáceres, Spain. Author: Alonso de Mendoza, year 2019. Reproduced with permission from Creative Commons. **(B)** Self-portrait of the Spanish painter Martín Rico, created in 1908, the year of his death. This image is in the public domain.

He passed away on September 21, 1558. Following the meal on August 30, 1558 [erroneously cited as August 31 in some sources (10)], the Emperor experienced a sudden sensation of coldness upon leaving the table, followed immediately by a heat that persisted for nearly 3 h (3). His butler, Luis Quijada, expressed his concern in a letter to Juan Vázquez de Molina, stating that ‘*it has been many years since His Majesty has had a fever with chills without a gout episode*’ (3). Despite undergoing bloodletting procedures twice, the emperor’s health deteriorated significantly, prompting him to request extreme rites. In the subsequent days, he completely lost his appetite for food (11). At the dawn of his passing, the Emperor uttered these words: ‘*It is time; bring me that candle and that crucifix*’. (10) After receiving them, he turned to the side and peacefully departed, pronouncing the words ‘*Oh, Jesus*’ (6, 10, 11). Numerous accounts indicate that the Emperor passed away while gazing upon Titian’s Last Judgment (2, 6) (Figure 2A).

Within the Monastery, there exists a wooden bench bearing an inscription dating back to the 16th century. The inscription states: ‘*His Majesty the Emperor D. Charles V, our lord, was seated in this spot when he was afflicted by the illness on August 31 at four o’clock in the afternoon. He departed from this life on September 21 at half past two in the morning. In the year of our Lord 1,558*’ (3).

In his testament of 1,554, Carlos V expressed his wish to be laid to rest in either Dijon or Granada. However, while residing in Yuste during his retirement, Carlos V penned a new will requesting to be interred alongside his wife, Isabel of Portugal, in Yuste or any other location except Granada. The ultimate decision regarding his final resting place was entrusted to his son, Felipe. Until a resolution was reached, Carlos V instructed for his temporary burial to take place beneath the main altar of the church in the Yuste monastery (2).

## 4 The body of Charles V and the investigation of the disease

The significance of the Emperor’s passing towards the end of September cannot be underestimated. Given the approaching winter season, his body was interred in a crypt located in the Spanish city of Cáceres, where temperatures frequently plummet below freezing. Consequently, instead of undergoing decomposition, the body underwent mummification. Adhering to the instructions outlined in Carlos V’s testament, his son Felipe II undertook the task of erecting a monastery as a tribute to his father and a final resting place for his remains. This ambitious undertaking materialized in the form of the Monastery of San Lorenzo de El Escorial (2).

In 1573, Felipe II of Spain, the son of the Emperor, convened his family at the Monastery of El Escorial in Madrid, Spain, to announce his decision to transfer his father’s remains to a vault within El Escorial. Subsequently, in 1574, the remains of Carlos V, his sister Leonor (interred in Mérida), Isabel of Portugal, Princess María of Portugal, and the infants Fernando and Juan (all buried in Granada) were brought to Yuste, with the intention of eventually transferring them to their ultimate resting place at the Monastery of El Escorial (2). There, they were interred together with the remains of María de Hungría, those of Isabel de Valois, and those of Prince Don Carlos, in the initial funerary chapel of the Habsburg family, within the crypt of the presbytery of the recently constructed Old Church (2). However, this would not be the final resting place for Carlos V’s body. In 1586, under the orders of Felipe II, the remains were transferred to the intermediate crypt located beneath the main altar of the basilica, serving as the ultimate pantheon (2).

**Figure 3:**
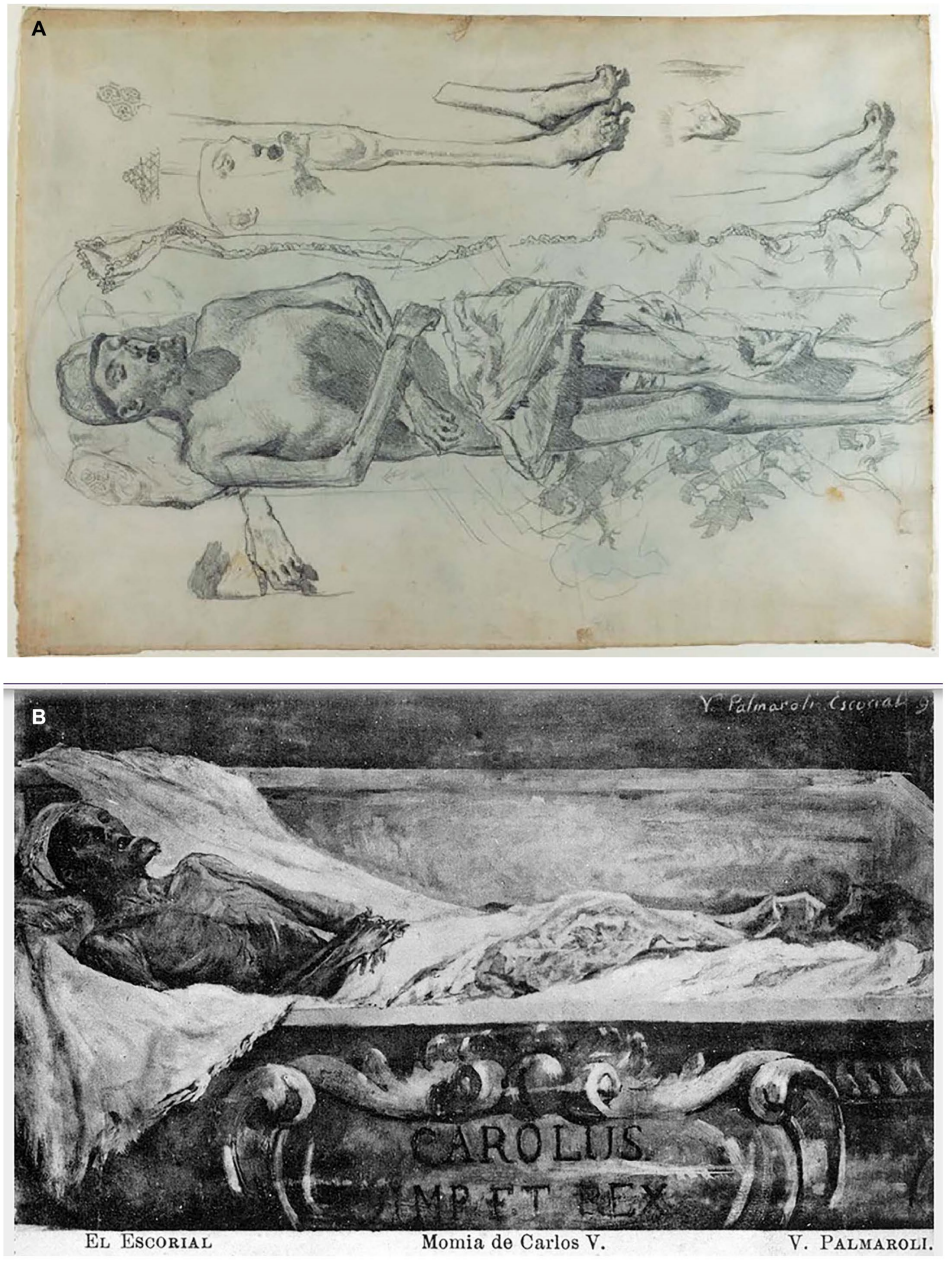
**(A)** Charles V in his sarcophagus, depicted by Martín Rico y Ortega in November 1871. **(B)** Charles V displayed in his sarcophagus. This postcard reproduces a painting from the years 1870–1872 by Vicente Palmaroli González.

In 1870, the revolution known as La Gloriosa erupted, leading to the abdication of Elizabeth II of Spain. A consequence of this revolution was the desecration of several tombs belonging to royalists, including that of Charles V. Remarkably, the emperor’s body had been remarkably preserved and mummified, with his beard remaining intact. This observation was made by the Spanish painter Martín Rico, who was tasked with documenting the state of the body (Figure 2B). Rico dedicated several days to sketching the beard (Figure 3A) and subsequently noted, ‘*I was struck by the fact that his thick beard, closely trimmed around the mouth, is dark brown and not gray, almost white, as depicted in the existing portraits of the valiant prince*.’

The revolutionaries made the decision to publicly exhibit the body for several days, initially without a lid, and later with a glass lid that could be lifted to touch the corpse. Eventually, a permanent glass lid was placed over the body to allow for contemplation without physical contact. Writer Pedro Antonio de Alarcón documented these events, stating, ‘*The tomb of Charles V was open, and in front of it, on a specially constructed scaffold, there stood a coffin without a lid, as it had been replaced by a glass cover of the same dimensions as the coffin. In the initial displays, there was no glass, or if there was, it would be lifted, enabling anyone who desired to pass their hand over the darkened face of the deceased*.’

In order to comprehend the extent of this “tourist” attraction, on December 9, 1870, the Spanish government extended an invitation to several foreign diplomats residing in Madrid, along with their families, to visit the exposed tomb at El Escorial. The visit was described by the British ambassador, Sir Arthur Layard, who remarked, ‘*The body is wrapped in white linen and red silk. A white linen cap embroidered with gold is placed upon his head. Some of the attendees claimed they could discern the features resembling Titian’s portrait, although this appeared to me to be an exaggeration. The only notable resemblance lies in the chin, which is highly distinctive and distinctly Austrian. It is adorned with a short red beard. The mummy is remarkably well-preserved. The hands and feet exhibit delicate proportions*.’ Sir Arthur Layard commissioned the painter Vicente Palmaroli y Rodríguez to create an oil sketch of the mummy, which was subsequently photographed and disseminated in the form of postcards (Figure 3B).

**Figure 4:**
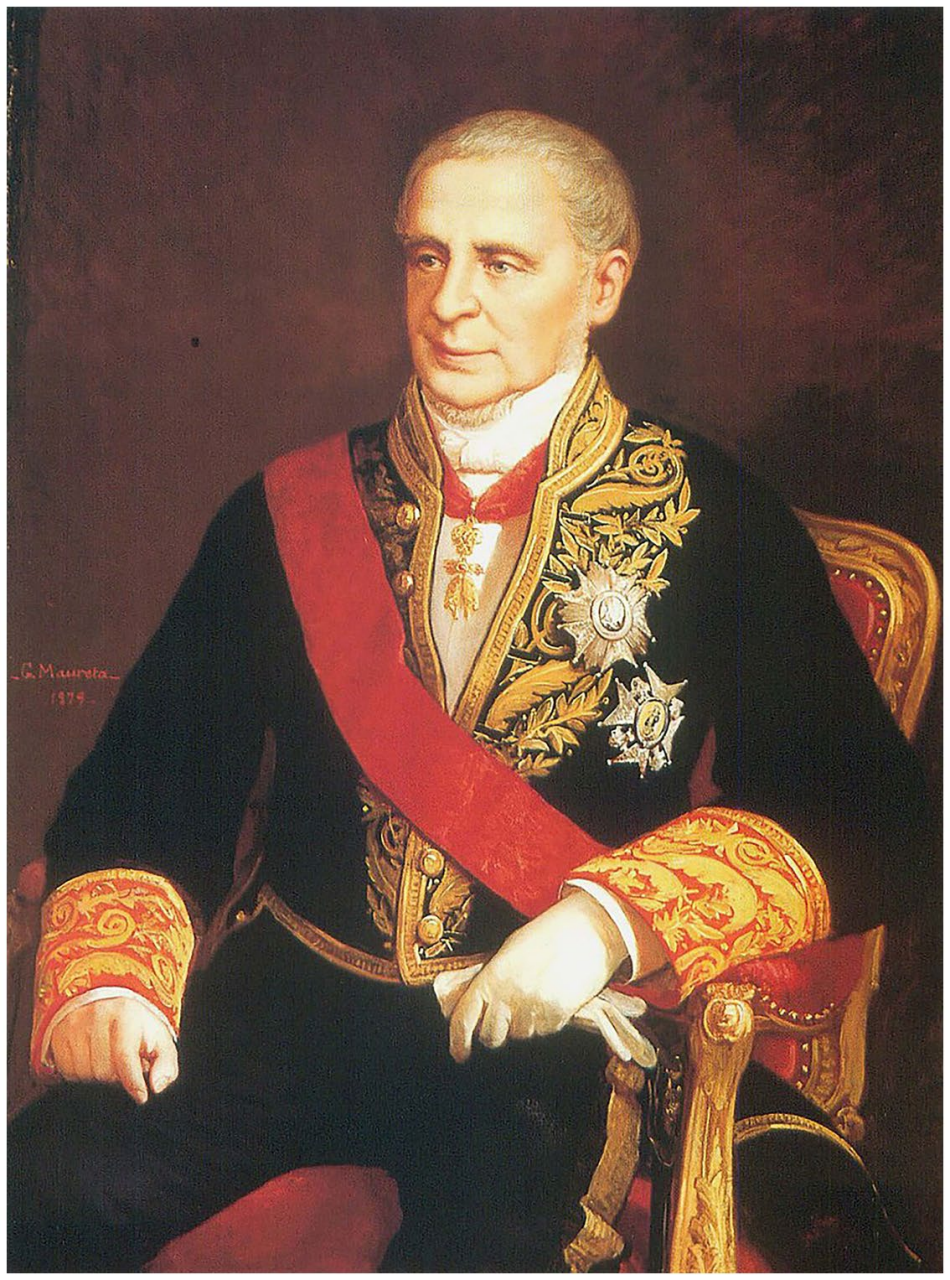
Portrait of Manuel Pando Fernández de Pinedo, Marquis of Miraflores and Pontejos, Spanish aristocrat, politician, and historian, Count of Villapaterna and Ventosa, Lord of Villagarcía del Pinar and Miraflores, Knight of the Order of the Golden Fleece, Knight of the Grand Cross of the Order of Charles III of Spain, and Knight of the French Legion of Honor and the Portuguese Order of Christ (1792–1872). The portrait was painted in 1879 by Gabriel Maureta Aracil. The image is in the public domain.

One of the visitors to the tomb, Don Manuel María de Pando y Fernández de Pinedo, the Fourth Marquis of Miraflores (Figure 4), developed a strong fascination with the mummy and desired to possess a relic from the body. In order to accomplish this, he offered a bribe of 20 *reales* (the currency of Spain at that time) to the crypt guard in exchange for a fragment of the emperor. The guard, succumbing to the bribe, severed the last phalanx of the little finger, which the Marquis retained as a memento from that moment onwards.

Years later, the Marquis of Miraflores would deeply regret his previous actions and confided in his sister, Genoveva de Samaniego y Pando, the widow of the Eleventh Marquis of Martorell (Alonso Tomás Álvarez de Toledo y Silva). Seeking guidance, she advised him to compose a letter, which they both signed, addressed to King Alphonse XIII of Spain. In this letter, dated September 14, 1870, they asserted that the acquisition of the finger had been ‘involuntary’ and that no ‘improper means’ had been employed in obtaining it. Upon reviewing the matter, Alphonse XIII made the decision to send the finger back to El Escorial, rather than interring it with the remainder of the emperor’s body. Consequently, the finger was placed within a red velvet urn in the Sacristy of the monastery, alongside other relics primarily associated with Catholic saints, and in proximity to the reinterred remains of the emperor.

There is limited information available regarding the veracity of the alleged second desecration of Charles V’s remains, which purportedly took place in 1936 during the Spanish Civil War by Republican militiamen. The international media extensively covered these grave violations and disseminated some of the most harrowing images captured during that time. Among these images is one depicting a militiaman embracing a mummy believed to be that of Charles V. Julian de Zulueta encountered this photograph in a French newspaper and asserted that he had identified the mummy as that of Charles V. According to Zulueta, ‘During our time in Paris, the Zuluetas, I had come across a photograph taken during the Civil War, which was published in the international media. In the photograph, a militiaman appeared playfully embracing the emperor’s mummy. The eyes of the mummy were open, as if one could almost expect it to utter words’ (12).

Julián de Zulueta y Cebrián was the nephew of the Spanish socialist leader Julián Besteiro. Following the establishment of the Spanish Republic, his father, Luis de Zulueta, was appointed as ambassador to Berlin, prompting the family to relocate to the German capital. This relocation took place around the time Adolf Hitler assumed the position of chancellor, approximately six months prior. Approximately one year after their arrival in Berlin, Zulueta personally witnessed the Night of the Long Knives and attended Hitler’s speech on the following day.

Zulueta was 18 years old when the Spanish Civil War broke out. The conflict found the Zuluetas in Rome, and from there, they made the decision to directly exile themselves to Colombia, which afforded Julián the opportunity to pursue his medical studies at the University of Bogota. Subsequently, he furthered his training at Cambridge and the London School of Tropical Medicine, specializing in tropical diseases. His career led him to join the World Health Organization in Geneva, Switzerland, embarking on a 30-year journey that took him to various countries including Malaysia, Uganda, Lebanon, Jordan, Syria, Iraq, Iran, Afghanistan, Turkey, Algeria, Pakistan, and Madagascar. During his travels, he spearheaded numerous campaigns against malaria. To this day, he is fondly remembered in Borneo as ‘*the Lord of the Mosquitoes*.’

Upon his return from exile, Zulueta established his residence in Spain and in 1983, he was elected as the socialist mayor of his hometown, Ronda. It was during this time that he came across an article discussing the potential rehydration of mummified tissues for the purpose of investigating diseases in deceased individuals (13). This, coupled with his profound interest in malaria, sparked the idea in Zulueta’s mind to demonstrate that Emperor Charles V’s death was caused by the disease. However, conducting the exhumation of the emperor’s remains necessitated the approval of the Spanish Royal House, then represented by John Charles I of the Bourbon dynasty. Regrettably, permission was not granted, thereby preventing Zulueta from proceeding with his investigation.

In 2004, Zulueta received information from the head of the National Heritage that the final phalanx of Charles V’s little finger had not been interred with the remainder of his body, but instead was placed in a small urn within the Royal Monastery of El Escorial. On this occasion, the Spanish Royal House kindly granted permission for the examination of the finger, a decision approved by John Charles I, the National Heritage, and the Ethics Committee of the Hospital Clínic in Barcelona, Spain.

Zulueta enlisted the expertise of Professor Pedro Alonso, who had extensive experience as the Director of the World Program against Malaria, and Dr. Jaume Ordi, a pathologist at the Hospital Clínic of Barcelona, for the undertaking. Additionally, he received assistance from Jordi Esteban, Martín Velasco, Ernest Mas, Elías Campo, and Pedro L. Fernández, all of whom were members of the Institut d’Investigacions Biomèdiques August Pi i Sunyer, affiliated with the Hospital Clínic and the University of Barcelona, Spain. The finger, still housed in its original urn adorned with red velvet, was transported to Barcelona under the escort of two Civil Guards, as befitting a reigning monarch.

The analysis of the finger unveiled that it pertained to the distal phalanx of the fifth finger, with an undetermined hand laterality. Its complexion exhibited a dark brownish hue and possessed a leathery texture. The proximal section of the nail plate seemed to be fused to the nail bed, whereas the distal part was entirely detached. A yellowish chalk-like residue completely obscured the proximal edge of the finger (14). Radiographic examination exposed pronounced erosion of the proximal epiphysis of the phalanx, characterized by uneven borders and calcifications within the soft tissues.

The specimen was subjected to rehydration through complete immersion in Sandison’s solution for a duration of 1 h. Following this, the most proximal segment (the joint’s epiphysis) was prepared for histological examination, generating sections for Hematoxylin–eosin, Masson’s trichrome, and Giemsa staining. The yellowish deposit was scrutinized using a scanning electron microscope and an X-ray microanalysis system. A segment of the deposit was also subjected to a reagent test, utilizing the Bayer Advia system from Bayer Diagnostics, for the identification of uric acid. This test relies on the enzymatic conversion of uric acid to allantoin and peroxide by uricase. The presence of a colored complex formed by hydrogen peroxide allows for a positive result. The sensitivity of this method is 0.1 mg/dL.

The histological sections revealed that the dermal collagen and bone exhibited excellent preservation, although no cells were found except for isolated red blood cells. In the proximal portion of the sample, the bone was replaced by a finely fibrillar basophilic deposit that extended into the surrounding soft tissues. Within these deposits, numerous acicular crystals were observed, exhibiting birefringence when examined under polarized light, which strongly indicated the presence of uric acid crystals. Electron microscopy further confirmed the presence of a deposit composed of acicular crystals that caused erosion and destruction of the bone. Lastly, chemical and colorimetric investigations conclusively determined that the deposit consisted of uric acid.

Consequently, the conducted studies unequivocally establish that the examined finger belonged to an individual afflicted with gout.

The emperor endured recurrent episodes of gout throughout his adult years, necessitating the construction of a custom chair for his mobility. The earliest recorded occurrence of a gout attack dates back to the year 1,528, while he was on a journey to Valladolid (9).

As early as 1,532, the emperor chronicled his gout episodes in a letter addressed to his daughter, Mary of Hungary. As the condition progressed, it not only impacted the joints of his feet but also affected his hands to such an extent that it hindered his ability to write letters.

In 1542, the emperor experienced his ninth gout attack, which affected a significant number of his joints and endured for a longer duration than any of the previous episodes. This ailment rendered him bedridden for several months. Subsequently, gout attacks became more frequent, accompanied by prolonged periods of incapacity and a gradual loss of joint mobility (9).

In one of his letters to Philip II, dated 1,553, the emperor expressed, ‘[…]*My dear daughter, I regret to inform you that this letter is not written by my own hand as the openings in my fingertip, which had almost healed, have reopened, causing me great pain* […] *I shall attend to the remainder of your letter tomorrow or the day after. Therefore, I will refrain from further elaboration in this correspondence* […]’ (9).

In this regard, the emperor would occasionally resort to the use of the Indian stick, seeking solace from his ailment, as documented by Luis de Ávila in his memoirs: ‘*Engaging in purgation with the Indian stick proved highly advantageous in alleviating his gout…*’ (9).

The investigations carried out by Juan de Zulueta encompassed not only the examination of the tophus in the little finger but also the determination of the Emperor’s cause of death, which was identified as malaria (15). In an article published in an Italian medical journal, Zulueta presented the findings of Giemsa staining conducted on rehydrated and fixed sections of the finger. The staining revealed the presence of ‘*small parasites exhibiting distinct bluish cytoplasm and red nuclei, likely originating from a ruptured mature schizont*’ (15). Zulueta concluded that ‘*while gout undeniably posed a challenge for the esteemed ruler, it was undoubtedly malaria caused by P. falciparum that led to his demise*’ (15).

The presence of *P. falciparum* in the finger tissue was further validated through molecular studies using PCR. This confirmation was provided verbally to the authors of this manuscript by Dr. Fernández (Department of Pathology, Hospital Universitario Germans Trias i Pujol, Badalona, Barcelona, Spain) and Dr. Ordi (Department of Pathology, Hospital Clínic Institut d’Investigacions Biomèdiques August Pi i Sunyer, University of Barcelona, Barcelona).

One final element was crucial to complete this entire narrative: confirming the finger’s rightful association with Charles V. The Spanish National Heritage had already affirmed its authenticity through official documentation. However, a DNA analysis could have definitively established the finger’s origin by comparing its genetic material with that of certain members of the House of Habsburg. Regrettably, permission to conduct the genetic study on the finger was withheld by the Spanish Royal Household.

## 5 Final discussion

The research on the Emperor’s illnesses, particularly regarding the cause of his death, consolidates several aspects in historical investigation. On one hand, it confirms the significance of studying Carlos V’s finger, demonstrating the successful histological examination of mummified remains. The histological images proved to be of optimal quality for histopathological interpretation through microscope examination, utilizing a processing procedure scarcely differing from that employed in routine biopsy studies in daily practice.

On the other hand, it reaffirms that the Emperor’s true cause of death was indeed malaria. These morphological findings added to the geographical, environmental, and epidemiological evidence mentioned in the text.

Finally, it substantiates, with histopathological evidence, the Emperor’s gout, a condition extensively documented in historical texts, letters, and portraits, now firmly confirmed by histopathological evidence.

## Data availability statement

The original contributions presented in the study are included in the article/supplementary material, further inquiries can be directed to the corresponding author.

## Ethics statement

Ethical approval was not required for the study involving humans in accordance with the local legislation and institutional requirements. Written informed consent to participate in this study was not required from the participants or the participants’ legal guardians/next of kin in accordance with the national legislation and the institutional requirements. Written informed consent was obtained from the individual(s) for the publication of any potentially identifiable images or data included in this article.

## Author contributions

AF-F: Conceptualization, Investigation, Methodology, Writing – original draft, Writing – review & editing. EF: Conceptualization, Project administration, Resources, Supervision, Writing – review & editing.
